# Procyanidins from Wild Grape (*Vitis amurensis*) Seeds Regulate ARE-Mediated Enzyme Expression via Nrf2 Coupled with p38 and PI3K/Akt Pathway in HepG2 Cells

**DOI:** 10.3390/ijms13010801

**Published:** 2012-01-13

**Authors:** Min-Ji Bak, Mira Jun, Woo-Sik Jeong

**Affiliations:** 1 Institute for Phytochemical-Drug Interactions, Department of Food & Life Sciences, College of Biomedical Science & Engineering, Inje University, Gimhae 621-749, Korea; E-Mail: redapplemj@hanmail.net; 2 Department of Food Science & Nutrition, Dong-A University, Busan 604-714, Korea; E-Mail: mjun@dau.ac.kr

**Keywords:** wild grape seed, *Vitis amurensis*, procyanidin, chemoprevention, MAPKs, Nrf2, phase II detoxifying enzyme, antioxidant enzyme

## Abstract

Procyanidins, polymers of flavan-3-ol units, have been reported to exhibit many beneficial health effects such as antioxidant and anti-carcinogenic effects. In this study, we investigated the cancer chemopreventive properties of procyanidins from wild grape (*Vitis amurensis*) seeds in particular their roles in inducing phase II detoxifying/antioxidant enzymes as well as in modulating the upstream kinases. Ethanolic extract of *V. amurensis* seeds was fractionated with a series of organic solvents and finally separated into six fractions, F1–F6. Chemical properties of the procyanidins were analyzed by vanillin assay, BuOH-HCl test, and depolymerization with phloroglucinol followed by LC/MS analysis. The F5 had the highest procyanidin content among all the fractions and strongly induced the reporter activity of antioxidant response element as well as the protein expression of nuclear factor E2-related factor (Nrf2) in HepG2 human hepatocarcinoma cells. The procyanidin-rich F5 also strongly induced the expression of the phase II detoxifying and antioxidant enzymes such as NAD(P)H:quinone oxidoreductase1 and hemeoxygenase1. Phosphorylations of the upstream kinases such as MAPKs and PI3K/Akt were significantly increased by treatment with procyanidin fraction. In addition, the procyanidin-mediated Nrf2 expression was partly attenuated by PI3K inhibitor LY294002, and almost completely by p38 inhibitor SB202190, but neither by JNK inhibitor SP600125 nor by MEK1/2 inhibitor U0126. Taken together, the procyanidins from wild grape seeds could be used as a potential natural chemopreventive agent through Nrf2/ARE-mediated phase II detoxifying/antioxidant enzymes induction via p38 and PI3K/Akt pathway.

## 1. Introduction

Chemoprevention is a cancer preventive approach using naturally occurring or synthetic chemical agents to block or reverse carcinogenesis [1]. Chemopreventive compounds are classified into blocking or suppressing agents based on their potential targeting of the carcinogenesis stages [2]. Blocking agents prevent carcinogens in the initiation stage by inducing detoxification of carcinogens undergoing their metabolic activation. Suppressing agents, on the other hand, inhibit the transformation of mutated cells into malignant tumor cells, in either the promotion or the progression stage [3]. Procyanidins, one of the natural chemopreventive agents, are the most abundant polyphenols in fruits, vegetables, cereals, green tea, nuts, seeds, bark and food grains and might decrease the risk of chronic diseases such as cardiovascular and cancers [4–10]. Procyanidins have received a great deal of attention over several decades, and many biological activities such as anti-oxidant [11,12], anti-mutagenic [13], anti-cytotoxic [6], anti-inflammatory [14] and cardioprotective effects [15] have been identified. Furthermore, procyanidins have been reported to inhibit lipid peroxidation [16], platelet aggregation [17] and to modulate the activity of disease-related molecular targets including COX-2 and nuclear factor κB (NF-κB) [18]. Mattito *et al*. have recently demonstrated the efficacy of diverse grape procyanidin fractions against oxidative damage induced by UVA and UVB radiation on human keratinocytes and epidermis [19]. These effects have been also confirmed by many animal studies, in which procyanidin inhibited tumorigenesis in tissues including skin [20,21], colon [21–23], breast [24] and prostate [25]. Therefore, procyanidins have been of great interest as a potential modulator for many molecular targets in chronic diseases including cancer.

*Vitis amurensis*, a wild-growing grape, is distributed mainly from the south-east regions of China to southern Korea. The fruit is not consumed fresh, but is used primarily for production of juice and wine due to its strong astringency. Its vine roots and leaves have been utilized in traditional Chinese medicine for the treatment of cancer and various types of pains [26]. An earlier study focused on the antioxidant properties of procyanidins fractions isolated from seeds of the wild grape [27]. Wang *et al*. described the presence of procyanidins in whole seeds of *Vitis amurensis*, but they did not elucidate the chemical structure of the active components [28]. Furthermore, some studies have suggested that the extracts from wild grape roots and leaves have anti-angiogenic activity [29], anti-inflammatory activity [30], and neuroprotective effects [31,32]. Our group has also reported that the 70% acetone extract form wild grape seeds and skins stimulates the Nrf2 in HepG2 cell [33]. However, the active compounds and molecular mechanism underlying the cancer preventive effects of wild grape seed has not been elucidated completely, although several hypotheses have been proposed.

Many studies have demonstrated that the molecular mechanisms and signaling pathway regulating the activation of NF-E2-related factor (Nrf2) as part of a cytoprotective mechanism are driven from its anti-carcinogenic activities [3,34,35]. Nrf2 is responsible for regulation of the anti-oxidant response element (ARE)-driven expression of genes encoding the majority of phase II detoxifying/antioxidant enzymes, such as NAD(P)H:quinone oxidoreductase-1(NQO1), glutathione S-transferases (GST), glutamate-cysteine ligase (GCL), and heme oxygenase-1(HO-1) [3]. Nrf2 knock-out mice are deficient in their ability to induce the phase II detoxifying enzyme, and as a result, are highly sensitive to oxidative stress and carcinogen-induced tumorigenesis compared with the wild-type mice [36].

A number of kinases are involved in this activation of Nrf2, which include mitogen-activated protein kinase (MAPK), protein kinase C, or phosphatidylinositol 3-kinase (PI3K) [35]. MAPK families including JNK, ERK and p38 are important upstream regulators of transcription factor activities and their signaling affects a wide variety of extracellular stimuli into intercellular events and thus controls the activities of downstream transcription factors implicated in carcinogenesis such as proliferation, differentiation, tumor promotion and apoptosis [37,38]. Kong *et al.* have shown that MAPK is involved in ARE activation and that is driven by Nrf2 dependent MAPK [39]. In addition, the PI3K/Akt signaling pathway plays an important role in a varied range of cellular processes to regulate the Nrf2/ARE pathway. A key downstream effect of PI3K is activation of serine-threonine kinase Akt, which in response to PI3K activation phosphorylates and regulates the activity of a number of molecular targets [40]. Several studies indicated that MAPKs and PI3K pathway have been implicated in the transcriptional regulation of the Nrf2 and phase II detoxifying/antioxidant enzymes [41,42].

In the present study, we sought to fractionate and isolate procyanidins from wild grape seeds, which were generated as wastes from food processing. In addition, we concentrated on the chemopreventive property relating their induction to Nrf2 expression and characterized the underlying mechanisms of Nrf2 regulation in the human hepatoma HepG2 cell line.

## 2. Results and Discussion

### 2.1. Flavan-3-ol Monomers in Fraction 4 (F4)

**Figure 1:**
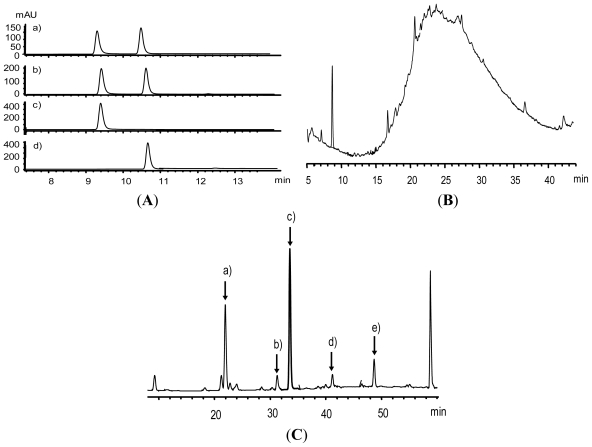
HPLC chromatograms detected by UV at 280 nm of (**A**) monomeric units isolated from fraction 4 of wild grape seeds eluted on Sephadex LH-20 column, (a) chromatogram of fraction 4 from wild grape seeds, (b) standard mixture of catechin and epicatechin, (c) catechin, (d) epicatechin; (**B**) procyanidin fraction (fraction 5) from wild grape seeds; (**C**) procyanidin fraction after phloroglucinol reaction (a) catechin-phloroglucinol adduct, (b) catechin, (c) catechin gallate-phloroglucinol adduct, (d) epicatechin, (e) epicatechin gallate.

To separate procyanidin fraction from wild grape seeds, we performed size exclusion chromatography over a Toyopearl HW-40F (Tosoh). F1–F4 were eluted with 50% aqueous MeOH to eliminate monomeric phenolic compounds and other small molecules except procyanidins. F5 was eluted by 66% aqueous acetone to obtain oligomeric and polymeric procyanidins and the last eluted by 100% acetone (F6). From HPLC-MS analysis of F4, we identified two monomeric flavan-3-ols, catechin and epicatechin. The data were confirmed by comparing retention times and molecular ion peaks with authentic compounds through a LC-MS system. The molecular ions *m*/*z* 291.5 were consistent with [M+H]^+^ of catechin or epicatechin (Figure 1A). The presence of monomeric flavan-3-ols in F4 might be responsible for the strong antioxidant activity and Nrf2/ARE inducing ability of F4.

### 2.2. Analysis of Procyanidins Fraction 5 (F5)

The interflavan bonds of procyanidins can be cleaved under acidic condition with high temperature, releasing extension and terminal subunits [43]. The released extension subunit intermediates (electrophiles) can be trapped by phloroglucinols (nucleophiles) to produce detectable adducts. The procyanidin fraction F5 from wild grape seeds was directly analyzed by HPLC and the hump in the chromatogram was implicated for the presence of polymeric procyanidins (Figure 1B). However, the depolymerization reaction of the procyanidin fraction with phloroglucinol generated several sharp peaks, suggesting break-down products from the reaction (Figure 1C). Compared with HPLC-MS chromatogram of authentic standard compounds, peaks 2, 4 and 5 were identified as catechin, epicatechin and epicatechin gallate, respectively; peaks 1 and 3 were identified as (*epi*)-catechin-phloroglucinol and epicatechin gallate-phloroglucinol, respectively. Mass data were also consistent with previously published data [43,44]. These results indicate that the major procyanidins of wild grape seeds consist of both prodelphinidins and procyanidins. According to peak area percents, the average degree of polymerization of procyanidins and prodelphinidins was 4.65 and 6.22, respectively.

### 2.3. Determination of Procyanidins (F5) from Wild Grape Seed Column Fractions

**Figure 2:**
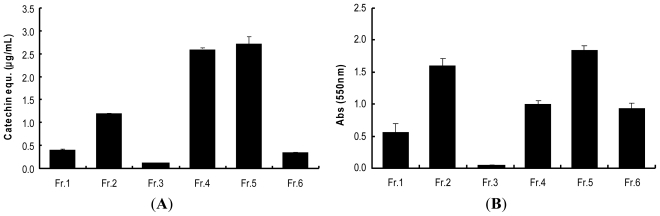
Determination of procyanidins from wild grape column fractions: (**A**) presence of terminal units; (**B**) presence of interflavan units obtained from the H_2_SO_4_ and BuOH-HCl assays. Values are means of three independent experiments ± SD (*n* = 3).

In order to determine the presence of procyanidins in wild grape seeds extracts and column fractions, the vanillin assay and BuOH-HCl hydrolysis method were employed. Procyanidins produce anthocyanidins through breaking interflavan bonds in acidic butanol solution (*n*-BuOH/HCl, 95:5) in the presence of iron (III) salts and heat (95 °C). As shown in Figure 2A of the six column fractions of wild grape seeds, fraction F4 and F5 were rich sources of flavan-3-ol terminal units probably due to presence of flavan-3-ol monomers and procyanidins, respectively. In BuOH-HCl assay of the column fractions, F5 was the richest source of compounds with interflavan units. Increased absorbance of F4 in this assay might result from the presence of anthocyanins in wild grape seeds and not from that of procyanidins (Figure 2B).

### 2.4. Procyanidins from Wild grape Seeds Induce ARE-Luciferase Reporter Gene Activity

**Figure 3:**
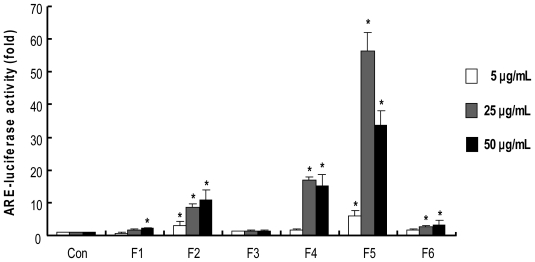
ARE-luciferase activity by wild grape seed column fractions. HepG2-C8 cells stably transfected with pARE-T1-luciferase reporter gene were treated with vehicle (DMSO, 0.1%) or each extract for 12 h. Luciferase activity was normalized with protein content and expressed as fold induction against vehicle-treated control. Values are means of four independent experiments ± SD (*n* = 4). * Significantly different from vehicle treatment, respectively (*p* < 0.05).

The induction of phase II detoxifying/antioxidant enzymes is largely mediated by the antioxidant response element (ARE), and the activation and/or induction of these enzymes is believed to be a crucial event for cellular defense against various carcinogens [3]. To determine whether ARE-mediated activities are affected by column fractions from wild grapes seeds, we used HepG2-ARE-C8 cells, which are stably transfected with the pARE-T1-Luciferase reporter gene using HepG2 cells [45]. As shown in Figure 3, treatment with F2, F4 and F5 showed stronger induction in ARE-luciferase activity than F1, F3 and F6. Especially, treatment with F5 at 25 μg/mL resulted in a tremendous induction of ARE-luciferase activity (about 55 fold compared to control), while treatment with F5 at 50 μg/mL showed a lower induction possibly due to cytotoxicity at the dose.

### 2.5. Induction of Nrf2 Protein Expression by Procyanidins from Wild Grapes Seeds in HepG2 Cells

**Figure 4:**
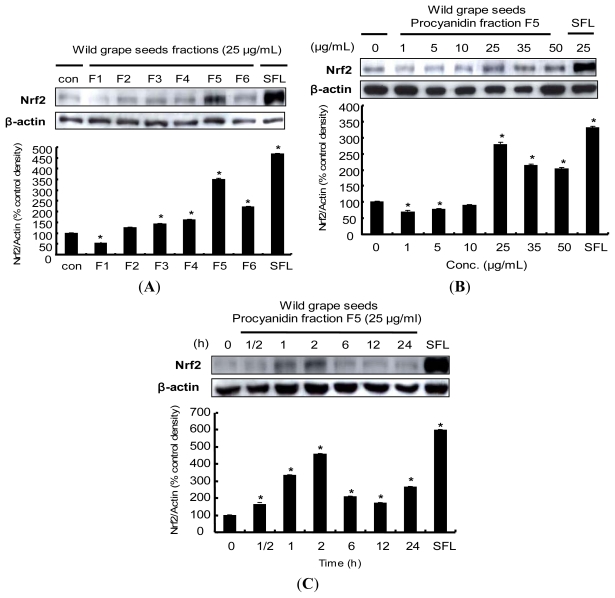
Effects of procyanidins from wild grape seeds on Nrf2 protein expression. (**A**) Nrf2 expression levels after treatments with wild grape seeds fractions. HepG2 cells were treated with 25 μg/mL column fractions for 2 h; (**B**) Nrf2 accumulation induced by procyanidin fraction F5. HepG2 cells were treated with F5 ranging from 1 to 50 μg/mL; (**C**) Time-dependent of Nrf2 accumulation after 25 μg/mL procyanidin fraction treatment. Samples were harvested at 0, 0.5, 1, 2, 6, 12 and 24 h time points. Induction fold of Nrf2 protein was calculated as the intensity of the treated samples relative to that of the control by densitometry. Sulforaphane (SFL) was used as a positive control. The blots shown are representative of three independent experiments with similar results. Values are means ± SD; *n* = 3, * *p* < 0.05 *vs.* control.

Nrf2 is a basic leucine transcription factors and plays an essential role in the ARE-mediated expression of phase II detoxifying/antioxidant enzymes such as NQO1, HO-1, GCL and GST [34]. These proteins may play a vital role in the prevention of cell dysfunction as a result of free radical production. Therefore, Nrf2 is considered to be an important potential molecular target for cancer prevention. To investigate whether wild grape seeds fractions (F1–F6) affect Nrf2 protein levels, we treated HepG2 cells with F1-F6 for 2 h. As shown in Figure 4A, F3, F4 and F6 slightly induced the accumulation of Nrf2 protein, while treatment with F5 dramatically increased the Nrf2 protein level. The F5 induced Nrf2 expression within the dose range of 1–50 μg/mL (Figure 4B). Next, Nrf2 protein expression was strongly induced after 2 h treatment and diminished after 6 h (Figure 4C). These observations were consistent with results from ARE luciferase activity, suggesting that the Nrf2 protein accumulation might contribute to the induction of ARE-mediated phase II detoxifying/antioxidant enzymes expression after treatments with wild grape seeds fractions, especially with F5. These findings are consistent with previous studies showing that certain phytochemicals and dietary plants exert potent chemopreventive activity through the induction of Nrf2:carnosol, a constituent of rosemary [46], sulforaphane [47], curcumin [48], diallyl sulfide [49] and quercetin [50]. In addition, many studies have identified Nrf2 induction in several different cells including human retinal pigment epithelial cells [51], human K562 cells [52], human lung epithelial cells [53], and PC12 cells [54].

### 2.6. Induction of the Nrf2-Driven Phase II Detoxifying/Antioxidant Enzyme Expression by Procyanidins from Wild Grape in HepG2 Cells

**Figure 5:**
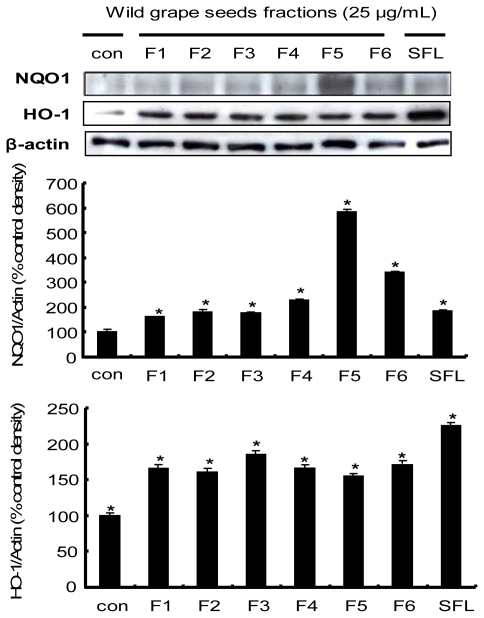
Effects of procyanidin from wild grape seeds on NQO1 and HO-1 induction in HepG2 cells. HepG2 cells were exposed to the 25 μg/mL of wild grape seeds fractions (F1–F6) for 24 h. The blots shown are representative of three independent experiments with similar results. Values are means ± SD; *n* = 3, * *p* < 0.05 *vs.* control.

We then took one step further to explore the molecular mechanisms underlying chemopreventive properties of procyanidins isolated from wild grape seeds, focusing on the up-regulation of phase II detoxifying/antioxidant enzymes including HO-1 and NQO1. Expression of phase II detoxifying/antioxidant enzymes has been known to be upregulated by the interaction between ARE in those genes and Nrf2, of which nuclear translocation iss stimulated by external and internal stimuli, such as phase II enzyme inducers [55]. HO-1 is a ubiquitous and redox-sensitive inducible protein that provides efficient cytoprotection against oxidative stress [4]. NQO1 is also generally considered to be a multifunctional antioxidant enzyme involved in cellular defense against electrophilic and oxidizing metabolites of xenobiotic quinones. NQO1 provides cell protection against radical damage, oxidative stress, or carcinogenesis [56]. F5 treatment significantly increased NOQ1 protein levels, suggesting the contribution of this enzyme to cellular antioxidant activity of wild grape seeds, while expression of the HO-1 protein was slightly increased by wild grape seeds fractions, implying an involvement of a different regulation pathway for HO-1 protein expression (Figure 5). These findings suggest that procyanidin isolated from wild grape seeds induced protein expression of NQO1 markedly, but less significantly for HO-1, which correlates with a noticeable increase in Nrf2 expression. Several studies have demonstrated that NQO1 up-regulation is mediated by activation of Nrf2-ARE pathway [50,52]. These results support the fact that the transcription factor Nrf2 is considered to be a key protein that up-regulates activity and expression of NQO1 through interaction with ARE.

### 2.7. Procyanidin-Induced Nrf2 Expression Is Associated with MAPKs and PI3K/Akt Pathways

**Figure 6:**
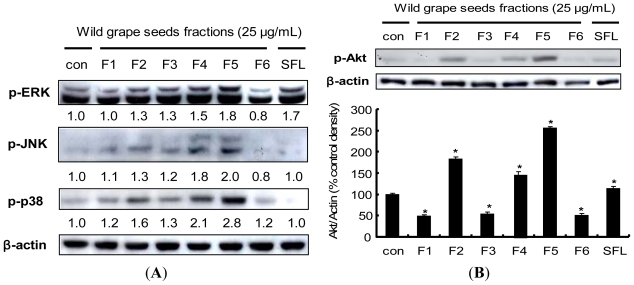

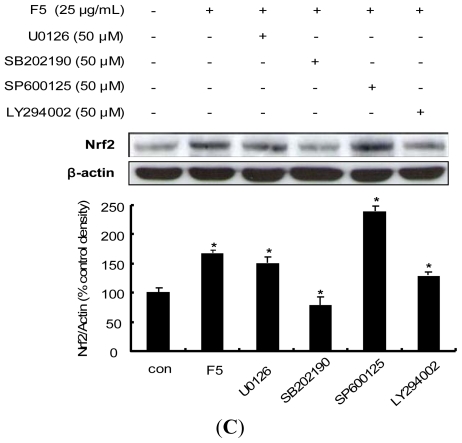
Effects of wild grape seed fractions on the phosphorylations of upstream targets in HepG2 cells. (**A**) Effect of wild grape seeds fractions on phosphorylation of MAPKs. Cells were treated with 25 μg/mL concentrations of wild grape seeds fractions (F1–F6) for 1 h; (**B**) Effect of wild grape seeds fractions on the phosphorylation of Akt. HepG2 cells were treated with vehicle (DMSO, 0.1%) or fractions and then equal amount of proteins from whole cell lysates were analyzed by Western blotting; (**C**) Influence of MAPK inhibitors on procyanidin-induced Nrf2 expression. HepG2 cells were incubated with 50 μM U0126 (MEK1/2 inhibitor), SB202190 (p38 inhibitor), SP600125 (JNK inhibitor) and LY294002 (PI3K inhibitor) for 1 h prior to 25 μg/mL procyanidin fraction treatment for 1 h. The blots shown are representative of three independent experiments with similar results. Values are means ± SD; *n* = 3, * *p* < 0.05 *vs.* control.

Induction of phase II detoxifying/antioxidant enzyme has shown that pathways involving MAPKs are responsible for the transduction of signals that initiate the gene activation [46,57]. To determine whether the Nrf2 expression induced by the procyanidins of wild grape seeds is associated with MAPK pathway, we examined the phosphorylation level of three MAPK subfamilies including JNK, ERK and p38. Phosphorylation levels of all three MAPKs were affected by treatment of wild grape seeds fractions (Figure 6A). Similar to the results shown in the previous figures, the procyanidin fraction (F5) was the most potent inducer of MAPKs phosphorylation. Sulforaphane elevated only phosph-ERK level (1.7 fold) whereas the procyanidin fraction F5 increased the expression of all three phospho-MAPKs. Several studies have demonstrated that activation of the MAPK pathway contributes to the induction of Nrf2 [27,34,41]. However, Li H *et al*. has recently revealed that Nrf2 activation in PC12 cells associated with ROS and the ubiquitin-proteasome pathway, not with MAPK signaling [4], suggesting cell type- and/or compound-specific regulation in Nrf2 signaling.

Activation of the upstream PI3K/Akt pathway and its contribution to the Nrf2-derived induction of phase II detoxifying/antioxidant enzymes by various polyphenol compounds have also been previously reported [46]. We therefore examined the effect of procyanidins from wild grape seeds on the phosphorylation of Akt. Figure 6B shows the increased level of phosphorylated Akt by wild grape seeds procyanidin fraction F5. Nrf2 expression has been associated with the PI3K/Akt pathway in several cell types: isoorientin, a major source of bamboo leaves, upregulates Nrf2 expression via the PI3K/Akt pathway in HepG2 cells [42]; capsaicin, a major pungent ingredient of red pepper, induces Nrf2 expression via activation of Akt in HepG2 cells [58]; the expression of Nrf2 by chlorophyllin is mediated via the PI3K/Akt pathways in human umbilical vein endothelial cells [59].

The involvement of these upstream kinases by wild grape procyanidins was further examined by application with their specific inhibitors; U0126 (a specific inhibitor of MEK1/2), SB202190 (a specific inhibitor of p38), SP600125 (a specific inhibitor of JNK) and LY294002 (a specific inhibitor of PI3K). Pretreatment with SB202190 completely blocked the procyanidin-induced expression of Nrf2, whereas SP600125 elevated the Nrf2 expression (Figure 6C). Pretreatment with LY294002 also attenuated the induced-Nrf2 expression by the procyanidins. These results indicated that the p38 might be involved in the procyanidin-induced Nrf2 expression, possibly through PI3K/Akt pathway. Similarly, curcumin accelerated Nrf2 expression via activation of PI3K and p38 MAP kinase in vascular smooth muscle cells [60], while induction of Nrf2 expression by resveratrol involves ERK pathway in PC 12 cells [54]. On the other hand, diallyl trisulfide (DATS), an organosulfur compound from garlic oil, has been reported to affect Nrf2/ARE activity via a calcium-dependent signaling pathway, but not by MAPK and PI3K pathways [61]. These evidences imply that expression of Nrf2 is modulated by various signaling pathways and also depending on the phytochemicals as well as on the cell types.

## 3. Experimental Section

### 3.1. Reagents

Wild grapes were kindly provided by Dooraemaeul Inc. (Hamyang, Kyongnam, Korea). (+)-Catechin, (−)-epicatechin, and phloroglucinol were purchased from Sigma (St. Louis, MO, USA). Solvents used for HPLC analysis were of HPLC grade from commercial sources. Antibodies against Nrf2, NQO1, β-actin polyclonal antibodies and peroxidase-conjugated anti-rabbit and anti-goat antibodies were purchased from Santa Cruz Biotechnology (Santa Cruz, CA, USA). HO-1 polyclonal antibody was purchased from Calbiochem (Darmstadt, Germany). Anti-phospho-ERK1/2, phospho-JNN, phospho-p38, phospho-Akt polyclonal antibodies, LY294002 (PI3 kinase inhibitor) U0126 (MEK1/2 inhibitor), SB202190 (p38 inhibitor) and SP600125 (JNK inhibitor) were purchased from Cell Signaling Technology (Beverly, MA, USA). All stock solution was dissolved in DMSO. The final concentration of DMSO in the culture medium was controlled to ≤0.1%. All the other chemicals were of analytical or molecular grade from commercial sources.

### 3.2. Extraction and Fractionation

Dried powder of wild grape seeds (5 kg) were extracted with 70% aqueous acetone at room temperature for 24 h, respectively. After suspension in water, 70% acetone extract of wild grape seeds was partitioned with n-hexane to remove hydrophobic compounds and chromatographed over a Toyopearl HW-40F (Tosoh, Tokyo, Japan) using an aqueous solution of 50% MeOH (F1–F4) and 66% acetone (F5), and 100% acetone (F6) to yield procyanidin fraction (Procyanidin fraction; F5). The fractions and compound were analyzed by comparing with authentic samples on a LC-MS system stated below.

### 3.3. LC-MS Analysis

LC-MS analysis was performed on an agilent 1100 liquid chromatograph system (Agilent Technologies Inc., Santa Clara, CA, USA) coupled to an in-line diode array detector (DAD), and an Agilent LC-MS mass spectrometer (PaloAlto, CA, USA) for the constituents determination of the wild grape seed fraction. C18 Column (5 μm, 4.6 × 250 mm, Phenomenex Inc., Torrance, CA, USA) was used for the HPLC. Mobile phase was consisted with water (A) and acetonitrile (B) as follows: 0 min, 10% B; 25 min, 60% B; 35 min, 100% B; 40 min, 100% B; 45 min, 0% B; 50 min, 10% B. Detection was set at 254 nm and 280 nm at ambient temperature.

### 3.4. Vanillin Assay

Procyanidin (for terminal units) contents of the fractions were determined by vanillin assay [43]. Briefly, samples were dissolved in minimum volume of methanol and filled up to 1 mL with glacial acetic acid. The sample solution reacted with 5 mL of vanillin reagent consisting of 1% vanillin and 8%. HCl in glacial acetic acid (1:1, v/v). The reaction mixture was placed in a water bath at 30 °C for 20 min exactly, and absorbance was read at 510 nm. Procyanidin contents of the fractions were expressed as (+)-catechin equivalents in mg per 100 g.

### 3.5. BuOH-HCl Hydrolysis

Procyanidin (for interflavan units) contents of the fractions were determined by BuOH-HCl hydrolysis, which were carried out by the method of Porter *et al* [62] with some modification. Briefly, 6 mL of acidic BuOH (5% (v/v) HCl in *n*-BuOH) and 200 μL of iron reagent (2% solution of FeNH_4_(SO_4_)_2_·12H_2_O in 2 N HCl) were added to 1 mg/mL of samples in test tubes with glass screw cap. After agitation, the tubes were heated at 95 °C for 50 min. The cooled reaction mixtures were measured absorbance at 550 nm. Quantification was performed by determining the average absorbance values.

### 3.6. Depolymerization of Procyanidins with Phloroglucinol

Depolymerization of wild grape procyanidins was carried out by a modified method from Kennedy and Jones [43] and Koupai-Abyazani *et al.* [63]. Procyanidins from wild grape (5 mg), phloroglucinol (50 mg), and ascorbic acid (10 mg) were dissolved in 1 mL 0.1 N HCl in MeOH. The mixture was shaken vigorously at 50°C for 20 min. After the reaction, the solvent was evaporated by nitrogen gas and dissolved in 500 μL of distilled water. The solution was extracted 5 times with 1 mL of ethyl acetate. The concentrated ethyl acetate extract was dissolved in 500 μL of 70% aqueous methanol and analyzed by LC-MS. The eluting solvents were (A) 1% aqueous acetic acid and (B) MeOH. The solvents A and B were as follows: 0 min, 5% B; 10 min, 5% B; 30 min, 20% B; 55 min, 40% B; 55.1 min, 90% B; 65 min, 90% B; 70 min, 5% B.

### 3.7. Cell Culture and Treatments

The human hepatoma cell line HepG2 was purchased from American Type Culture Collection (ATCC, Rockville, MD, USA). Cells were cultured at 37 °C, humidified at an atmosphere of 95% and 5% CO_2_ in F-12 medium supplemented with 10% FBS, 100 units/mL penicillin, 100 μg/mL streptomycin, 1% essential amino acids and, 0.1% insulin. Cells were seeded on 6-well plates at a density of 1 × 10^5^ cells/well for each experiment and allowed to grow for 24 h. Cells were seeded on plates and allowed to grow for 24 h. Then, cells were starved overnight with serum-free F-12 media prior to further treatments with either vehicle (DMSO, 0.1%) or various concentrations of wild grape seeds fractions.

### 3.8. ARE-Reporter Gene Activity

HepG2-C8 cell line was kindly donated by Dr. Ah-Ng Tony Kong (Rutgers University, Piscataway, NJ, USA), which was established by the stable transfection of HepG2 cells with *p*-ARE-T1-luciferase reporter gene as previously described [45,64]. The Nrf2-mediated ARE luciferase assay stabilized in HepG2-C8 cell was used to investigate the potential of the wild grape seeds fractions in activating the Nrf2/ARE signaling pathway. The HepG2-C8 cells were treated with fractions of wild grape seeds for 12 h. The ARE-luciferase activity was determined using a luciferase kit from Promega (Promega Corp., Madison, WI, USA) according to the manufacturer’s instructions. After treatments, cells were washed twice with ice-cold phosphate buffered-saline (pH 7.4) and harvested in 1× reporter lysis buffer. After centrifugation at 13,000 rpm for 10 min, a 10 μL aliquot of the supernatant was assayed for luciferase activity with a GloMax luminometer (Promega). The luciferase activity was normalized against protein amount, determined by a BCA protein assay (Pierce, Rockford, IL, USA), and expressed as fold of induction over luciferase activity of control vehicle-treated cells.

### 3.9. Western Blot Analysis

After treatment, cells were washed twice with ice-cold PBS (pH 7.4) and harvested using a cell scraper and lysed in 1× whole cell lysis buffer supplemented with protease inhibitors (Complete Cocktail; Roche Molecular Biochemicals, Indianapolis, IN, USA). The cells pellets were resuspended in lysis buffer on ice for 1 h, and cell debris was removed by centrifugation. The protein concentrations were determined using the BCA protein assay (Pierce) reagent according to the manufacturer’s instructions. Equal amounts of proteins were loaded onto a 10% SDS-polyacrylamide for gel electrophoresis and then transferred onto a PVDF membrane for 1 h using a semidry transfer system (Bio-Rad, Hercules, CA, USA). The membrane was blocked with 5% nonfat milk in PBST at RT for 1 h, and then incubated with primary antibodies overnight. After hybridization with primary antibody, the membrane was washed five times with PBST for 5 min, incubated with anti-goat or anti-rabbit IgG horseradish peroxidase-conjugated secondary antibodies and visualized using Western Blotting Luminol Reagents (Santa Cruz Biotechnology).

### 3.10. Statistical Analysis

Data were expressed as means ± standard deviation (SD). Statistical analyses were performed using SigmaPlot 8.0 software (Systat Software Inc., Chicago, IL, USA). Values were compared to vehicle controls by and unpaired Student’s *t*-test. *p* < 0.05 was considered as significant.

## 4. Conclusions

In conclusion, our study addresses the separation and chemopreventive properties of procyanidins from wild grape seeds. Here, we report for the first time that the major procyanidins of wild grape seeds consist of both prodelphinidins and procyanidins with an average degree of polymerization of 4.65 and 6.22, respectively. The wild grape seed procyanidins strongly induce Nrf2 expression, ARE-mediated transcription activation and thereby activate the expression of the Phase II/antioxidant enzymes, possibly via PI3K/Akt and p38 pathways in HepG2 cells. Taken together, our results suggest that procyanidins of wild grape seeds might be a new potential source of natural chemopreventive agents.
